# Reproducibility of smooth muscle mechanical properties in consecutive stretch and activation protocols

**DOI:** 10.1007/s00424-025-03075-7

**Published:** 2025-03-22

**Authors:** Simon Kiem, Stefan Papenkort, Mischa Borsdorf, Markus Böl, Tobias Siebert

**Affiliations:** 1 Department of Sport and Movement Science, University of Stuttgart, Stuttgart, Germany; 2 Institute of Mechanics and Adaptronics, Technische Universität Braunschweig, Brunswick, Germany; 3 Stuttgart Center for Simulation Science, University of Stuttgart, Stuttgart, Germany

**Keywords:** Adaptation, Urinary bladder, Stimulation, Stress–strain-relationship, Biological soft tissue

## Abstract

Mechanical organ models are crucial for understanding organ function and clinical applications. These models rely on input data regarding smooth muscle properties, typically gathered from experiments involving stimulations at different muscle lengths. However, reproducibility of these experimental results is a major challenge due to rapid changes in active and passive smooth muscle properties during the measurement period. Usually, preconditioning of the tissue is employed to ensure reproducible behavior in subsequent experiments, but this process itself alters the tissue’s mechanical properties. To address this issue, three protocols (P1, P2, P3) without preconditioning were developed and compared to preserve the initial mechanical properties of smooth muscle tissue. Each protocol included five repetitive experimental cycles with stimulations at a long muscle length, varying in the number of stimulations at a short muscle length (P1: 0, P2: 1, P3: 2 stimulations). Results showed that P2 and P3 successfully reproduced the initial active force at a long length over five cycles, but failed to maintain the initial passive forces. Conversely, P1 was most effective in maintaining constant passive forces over the cycles. These findings are supported by existing adaptation models. Active force changes are primarily due to the addition or removal of contractile units in the contractile apparatus, while passive force changes mainly result from actin polymerization induced by contractions, leading to cytoskeletal stiffening. This study introduces a new method for obtaining reproducible smooth muscle parameters, offering a foundation for future research to replicate the mechanical properties of smooth muscle tissue without preconditioning.

## Introduction

Organ models are crucial for understanding organ function [29] and for clinical applications, such as predicting surgical procedures and disease effects [11]. For a realistic representation of organ function, these mechanical models require accurate material parameters. Typically, the determination of material parameters as input data for mechanical muscle models requires the execution of multiple experiments involving muscle stimulation at varying lengths [6, 20, 32, 35]. Determining these parameters is particularly difficult for smooth muscle (SM) tissue due to the rapid changes in active and passive properties caused by cellular adaptation [46]. Furthermore, the reproducibility of experimental test results is a pressing challenge in current SM research [44]. The aim of this study is to reproduce the initial mechanical properties of SM tissue after stretch- and activation-induced changes in muscle structural properties using appropriate experimental protocols.

Microstructurally, SM lacks the striation pattern seen in skeletal muscle due to the absence of parallel myofibrils and Z-discs separating sarcomeres [1, 38, 43]. SM cells (SMC) are spindle-shaped with a central nucleus and are several hundred micrometers long and 5–6 µm wide when fully relaxed [1]. An SM sarcomere is composed of thick (myosin) and thin (actin) filaments, as well as so-called dense bodies that anchor the actin filaments. Additionally, the dense bodies serve as attachment points for the intermediate filaments, which form an elastic cytoskeleton within the cell. During contraction, myosin heads attach to actin, followed by the force-generating power stroke [1, 9, 33].

A number of studies have demonstrated that activation [15, 19, 45], passive stretch amplitude [47], and resting time [48] result in changes in active force within minutes to hours. Wang et al. [48] observed that passive stretching and shortening of rabbit tracheal SM over a 24-h period resulted in changes in active force and the optimal length (*L*_opt_) shifted as a consequence. Starting from an initial in situ length, passive stretching of tissue strips shifted *L*_opt_ to longer lengths, whereas passive shortening shifted *L*_opt_ to shorter lengths. In addition, the active force increased when multiple isometric contractions were performed at the same length. The authors proposed that these changes in *L*_opt_ are the result of an adaptive response by the SM tissue by adjusting the number of contractile units in series to maintain an optimal overlap of filament pairs [22, 48]. Increased SM forces are possible by increasing the number of contractile units in parallel [19].

The idea of a malleable myofilament lattice in an SMC, whereby sarcomeres are added and removed, is supported by several sources [4, 19, 22]. In SMCs, there are pools of free myosin monomers as well as filamentous myosin integrated into sarcomeres [8]. These pools are under constant remodeling, e.g., in response to activations [8]. Myosin monomers are added to the contractile units to adapt to longer lengths and removed from them to adapt to shorter lengths [4, 8, 46]. Smolensky et al. [37] showed that when the SM tissue is stimulated at long lengths, an increase in birefringence occurs within seconds, indicating an increased mass of thick filaments (myosin). During relaxation and shortening, myosin filaments partially dissociate and become free myosin monomers again. These observations indicate that the contractile apparatus in SMCs undergoes remodeling to maintain optimal overlap of contractile elements, ensuring the necessary contractile properties after large length changes [8, 34, 46].

In addition to the observed changes in active forces, Speich et al. [40] reported changes in the passive force–length relationship and a reduction in the maximum passive force of the rabbit detrusor muscle for repeated stretches combined with intermediate contractions at different lengths. Additionally, repeated stretches resulted in an increase in slack length [5] and changes in the curvature of the force-strain relationship [27, 31]. Interestingly, inducing an isometric contraction at short lengths appears to restore the passive forces [5, 40] and the slack length [5].

Preconditioning is a widely used method to adjust the adaptation and reproducibility of measured mechanical parameters during experiments. This method is used to ensure that the SM tissue reaches an “equilibrium state” where passive stress-stretch curves or active force responses are stable and repeatable, allowing the determination of the tissue’s mechanical properties [27]. Typically, SM tissue strips are cyclically stretched approximately 10 times during preconditioning [7, 14, 21, 28, 49]. This results in a reduction of passive forces and hysteresis, as well as an increase in resting length [27, 44]. Thus, the muscle parameters obtained after preconditioning do not correspond to the initial tissue response. Therefore, there is a gap in the literature regarding reproducible mechanical parameters without preconditioning that characterizes the initial mechanical state.

## Materials and methods

### Tissue handling and preparation

This study was exempted from ethical committee review in accordance with national regulations (German Animal Welfare Act), as urinary bladders from nine healthy, female domestic pigs (Sus scrofa domestica, age: ≈ 6 months, mass: ≈ 100 kg) were obtained from a slaughterhouse immediately after animal sacrifice. During transport to the laboratory, the bladder tissue was maintained in a modified Krebs solution (25 mM NaHCO_3_; 1.2 mM NaH_2_PO_4_; 2.4 mM MgSO_4_; 5.9 mM KCl; 2.5 mM CaCl_2_; 117 mM NaCl; and 11 mM C_6_H_12_O_6_) [24] at a constant temperature of 4°C [26]. During the experiments, the solution was maintained at a temperature of 37 °C and was aerated with 95% O_2_ and 5% CO_2_ (pH 7.4) [3]. The experiments were completed within 14 h on each day of testing. The bladder was cut open along the *ligamentum vesicae medianum*, and three strips were excised in longitudinal direction from the body region of each bladder. This resulted in a total of 27 tissue strips (*N* = 27) with a mean slack length (*L*_*S*_) of 10.4 ± 1.7 mm and a mean cross-sectional area (CSA) of 18.9 ± 4.5 mm^2^. After the measurements, the muscle tissue of the tissue strip was first separated from the serosa and then weighed. We determined the CSA as

$$CSA= \frac{m}{{\rho L}_{S}}$$

where *m* is the sample mass, $\rho$ = 1.05 g/cm^3^ is the SM density [18], and *L*_*S*_ is the sample slack length.

**Fig. 1:**
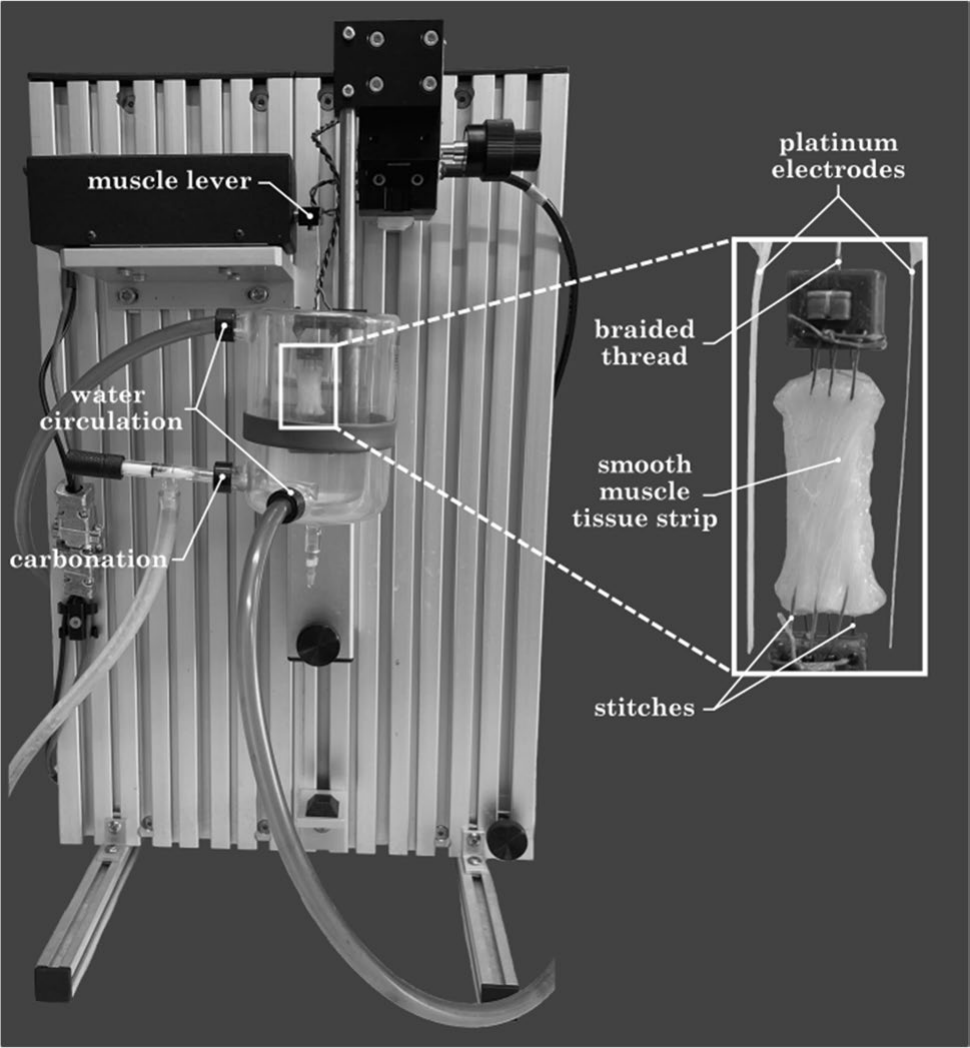
The illustration shows the experimental setup of the *Aurora Scientific* muscle lever system. To the right is a detailed image of a sutured SM strip. The upper end of the strip is connected to the muscle lever by a braided fishing line, which is used to induce stretch and record force. Muscle contractions are induced by electrical stimulation via platinum electrodes placed laterally along the strip. The tissue strip shown here is ~ 18 mm long. The graphic is for demonstration of the experimental setup and the strip shown is not included in the sample size. Figure adapted from Borsdorf [2]

For the purpose of tissue testing, a setup was used that consisted of a muscle lever system (305C-LR, *Aurora Scientific*, Canada) and a real-time software package (610A Dynamic Muscle Analysis, *Aurora Scientific*, Canada) for data acquisition. The length and force signals from the lever were recorded at 100 Hz using an analog-to-digital A/D interface (604A, *Aurora Scientific*, Canada). The tissue strips were sewn into 3D-printed mounting parts and then attached vertically to the muscle lever (Fig. 1). While the strips were fixed in the setup, they were held at a very short length (clearly sagging) to prevent any pull on the tissue. The slack length (*L*_*S*_) was then adjusted by manually stretching the strip until a force of 10 mN was reached. The exact *L*_*S*_ was then measured with a digital sliding caliper. The respective protocol was then initiated.

### Tensile testing protocols

**Fig. 2:**
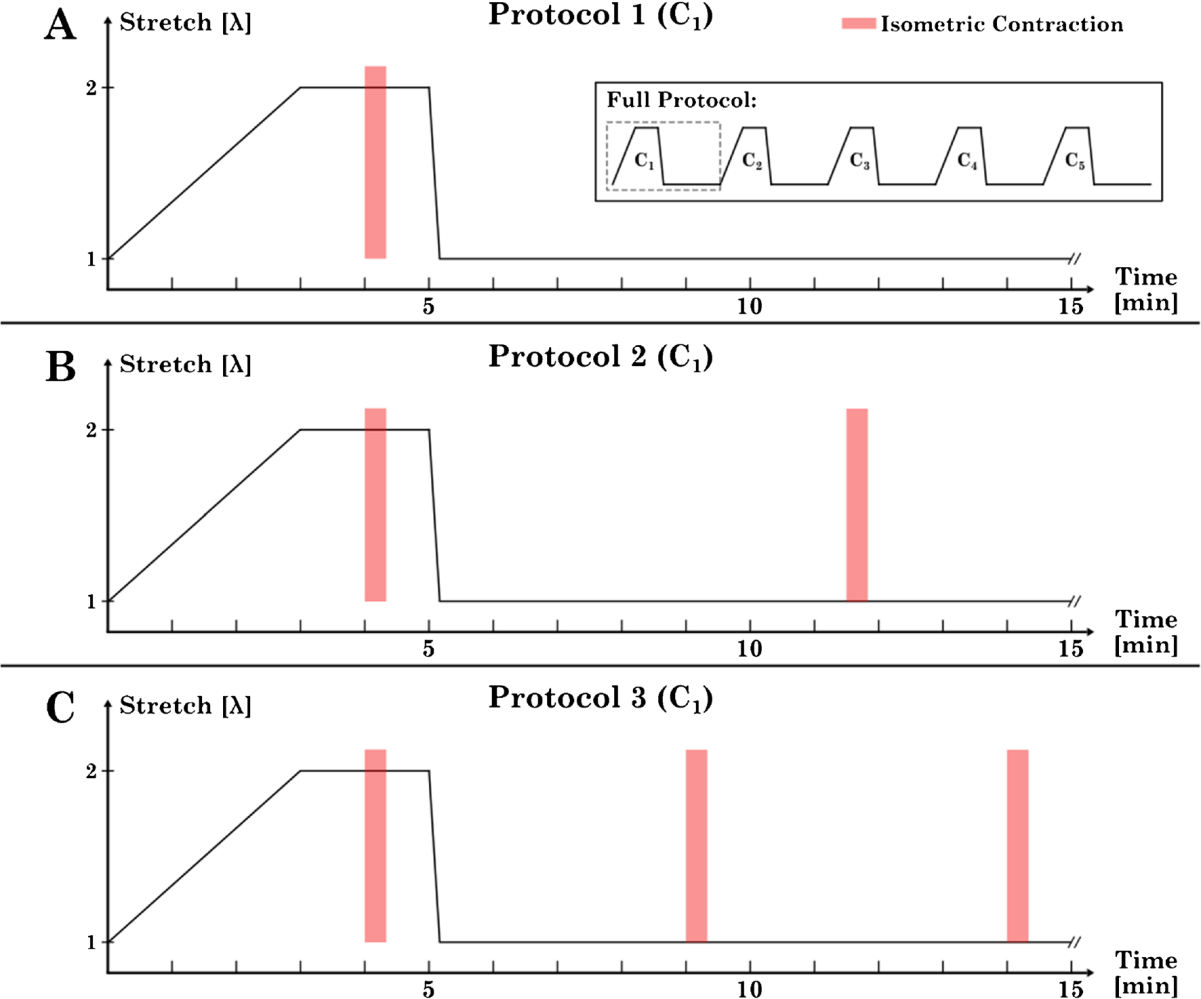
Schematic representation of the stretch-time curves of the three developed adaptation protocols. The sub-figure in **a** shows the full protocol that each tissue strip was subjected to (cycles C_1_ to C_5_). The first (C_1_) of the five repetitive cycles for each protocol is shown for each protocol: **a** Protocol 1 (P1), **b** Protocol 2 (P2), and **c** Protocol 3 (P3). The red bars indicate the isometric stimulation times (20 s each). *L*_*S*_ corresponds to λ = 1

In total, three different protocols have been developed (Fig. 2). All three protocols share the same basic structure, starting from *L*_*S*_ (corresponding to λ = 1). At the beginning, a length ramp was induced, stretching the strip to a stretch of λ = 2 at a strain rate of 1/180 [− /s]. The strip was then held at this length for 1 min. Subsequently, an isometric contraction (Fig. 2, first red bar from the left) was induced for 20 s, followed by a 40 s pause. Subsequently, the strip was shortened back to *L*_*S*_ at a rate of 1/10 [− /s]. The three protocols differ in the number of stimulations at *L*_*S*_.

For protocol 1 (P1), the strip was maintained at λ = 1 for 10:20 min until the next length ramp started (Fig. 2a). Therefore, no stimulation was induced at the short length. This cycle was repeated five times (designated C_1_–C_5_). The second protocol (P2 – Fig. 2b) included an additional isometric contraction at λ = 1 (Fig. 2b, second red bar from the left), timed precisely halfway between the contractions at λ = 2. The third protocol (P3 – Fig. 2c) included a third isometric contraction, again at λ = 1 (Fig. 2c, third red bar from the left). The protocols were designed with a minimum pause of at least 5 min between contractions to allow the tissue to fully recover [3, 30, 42, 48].

Muscle contractions during the protocols were induced by electrical stimulations (900 mA, 100 Hz, 5 ms) according to van Mastrigt and Glerum [24] for 20 s. From each bladder, the three strips were each assigned to one protocol, resulting in nine strips (*n* = 9) per protocol. The order of the assigned protocols was randomized for each bladder to avoid systematic errors in the results due to the storage time of the tissue strips.

### Analyzed mechanical parameters

**Fig. 3:**
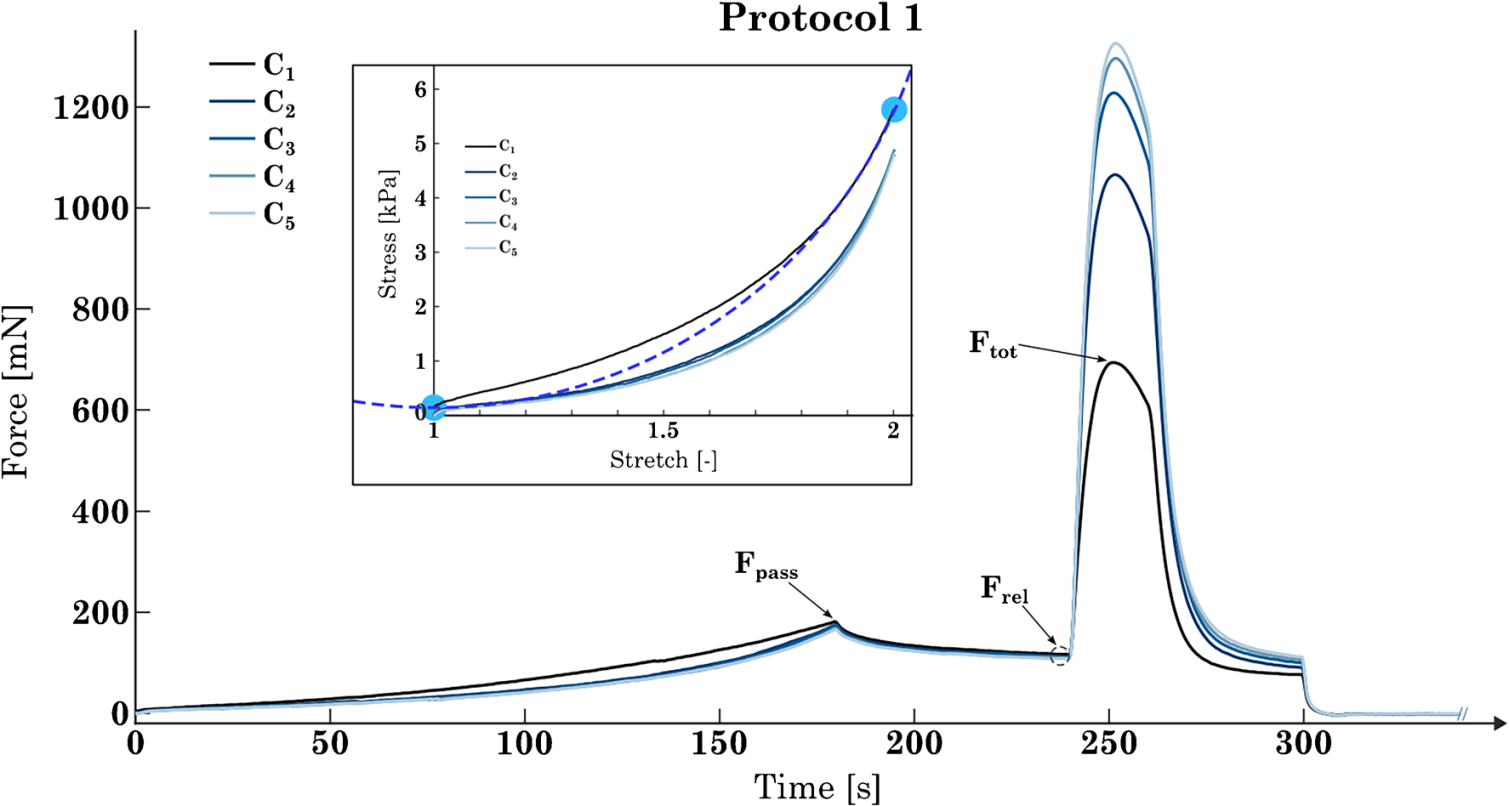
Exemplary force–time curves of lengthening ramps from λ = 1 to λ = 2 (see Fig. 2a) and subsequent isometric contractions for the repetitive 5 cycles. Note that the force responses of the 5 cycles are superimposed. The first cycle (C_1_) is shown in black, while the following cycles C_2_ to C_5_ are shown in various shades of blue. *F*_act_ is the force difference between *F*_tot_ (total force) and *F*_rel_ (force value after the relaxation phase of the tissue strip just before the induced contraction). *F*_pass_ is the passive force at the end of the lengthening ramp. The sub-figure shows a schematic example of how the circle segment (dotted line) was fitted using the start and endpoints (blue dots) of the ramp’s stress-stretch curve as anchor points. In this case, the circle was fitted to C_1_ (black line)

To assess the adaptation of SM tissue to the different protocols (P_1_, P_2_, P_3_), three parameters were determined. The first parameter was the maximum active force (*F*_act_) generated during the isometric contractions at λ = 2. *F*_act_ was calculated by subtracting the passive force during the stress relaxation just before the start of stimulation (*F*_rel_) from the maximum total force during stimulation (*F*_tot_) (Fig. 3). The second parameter observed was the maximum passive force (*F*_pass_) at the end of the lengthening ramp (Fig. 3). The final parameter was the curvature of the normalized passive stress–strain response during the lengthening ramps. For this purpose, a circle was fitted to the stress–strain curve of each ramp using least-squares fitting. Therefore, the endpoints of the circle fit had to align with the endpoints of the stress–strain curve. The curvature was then calculated as the inverse of the radius of the circle. Because for the curvature calculation process, the stress–strain curves were normalized with respect to stress and strain, the resulting curvature values are dimensionless and are therefore presented without units.

### Data processing and statistics

Smoothing of the raw data and further data analysis were conducted using Matlab (version R2023a Update 2, *The MathWorks Inc.*, Natick, MA, USA). The data presented in this study are presented as mean ± standard deviation. For the statistical analysis, the parameters were normalized with respect to the first cycle (C_1_). An ANOVA was conducted to test for significant differences between the protocols at C_5_. *Post-hoc* analyses were conducted using the Bonferroni correction. To test for differences within each protocol, a regression analysis was conducted. First, a regression line was constructed for each protocol from the mean values for the cycles 1–5 (Fig. 3). *T*-tests were then used to test the null hypothesis for the slope of the linear regression line, namely whether the slope differed significantly from a slope of zero. All analyses were conducted with a significance level of *p* < 0.05. The ANOVA and the *t*-tests were conducted using SPSS 23 (version 29.0.1.1, *IBM Corp.*, Armonk, NY, USA), while the regression analysis was conducted with Matlab.

## Results

**Fig. 4:**
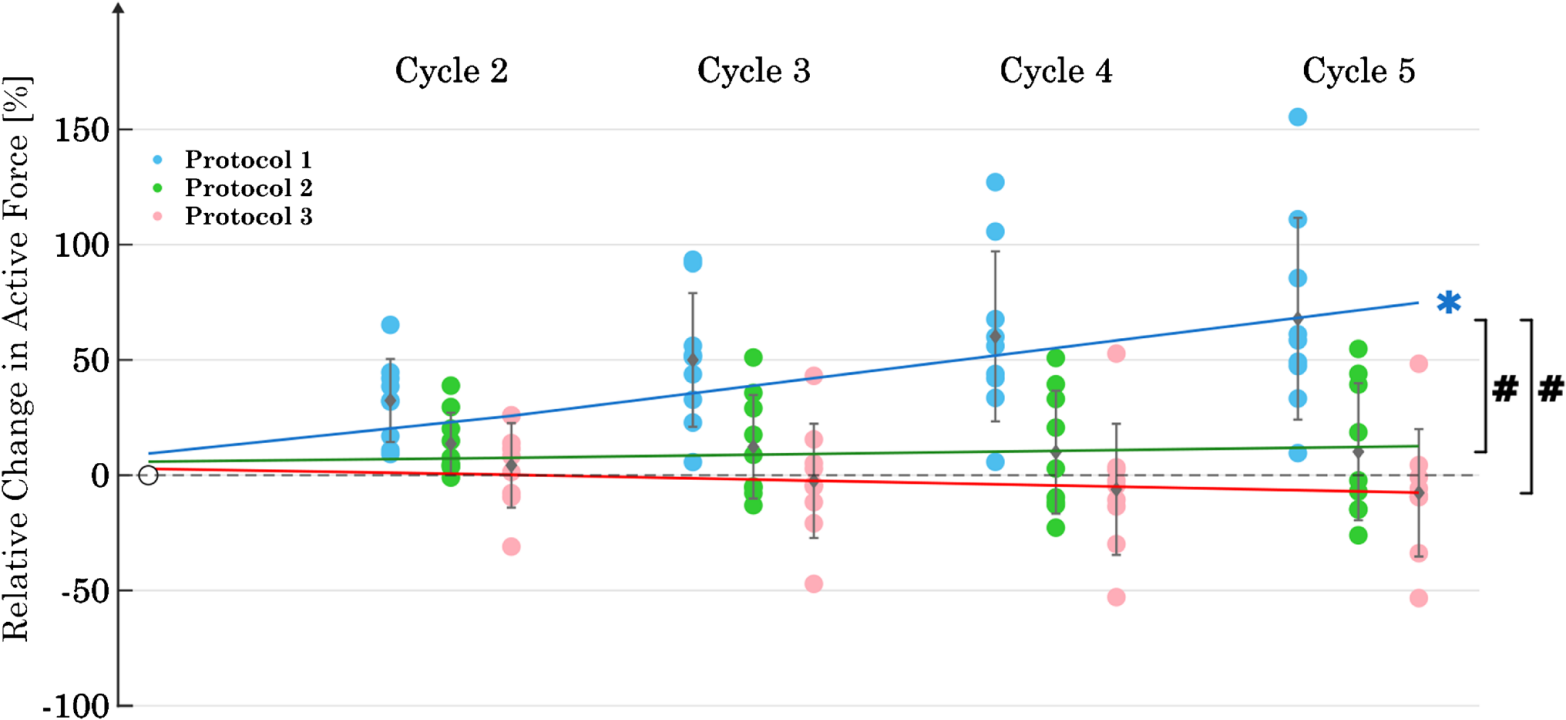
Change in active forces at λ = 2 over 5 cycles for each of the three protocols (*n* = 9). The data have been normalized to the value of the first cycle. The asterisk symbol (*) indicates a statistically significant trend (*p* < 0.05) in the regression analysis within each protocol for the slope of C_1_–C_5_. The number sign symbol (#) shows a statistically significant difference between the protocols with regard to the values at C_5_

For P1, the active forces increased with each cycle (Fig. 4). The mean active stress during C_1_ was 35.0 ± 24.4 kPa for P1, 34.5 ± 17.3 kPa for P2, and 38.9 ± 17.4 kPa for P3. Regression analysis revealed a statistically significant increase in slope for P1, with *p* < 0.001. For both P2 and P3, no significant trend in slope was observed. When comparing the values at C_5_, the results of the ANOVA showed a significant difference in active force between P1 and P2 (*p* = 0.005) and between P1 and P3 (*p* < 0.001).

**Fig. 5:**
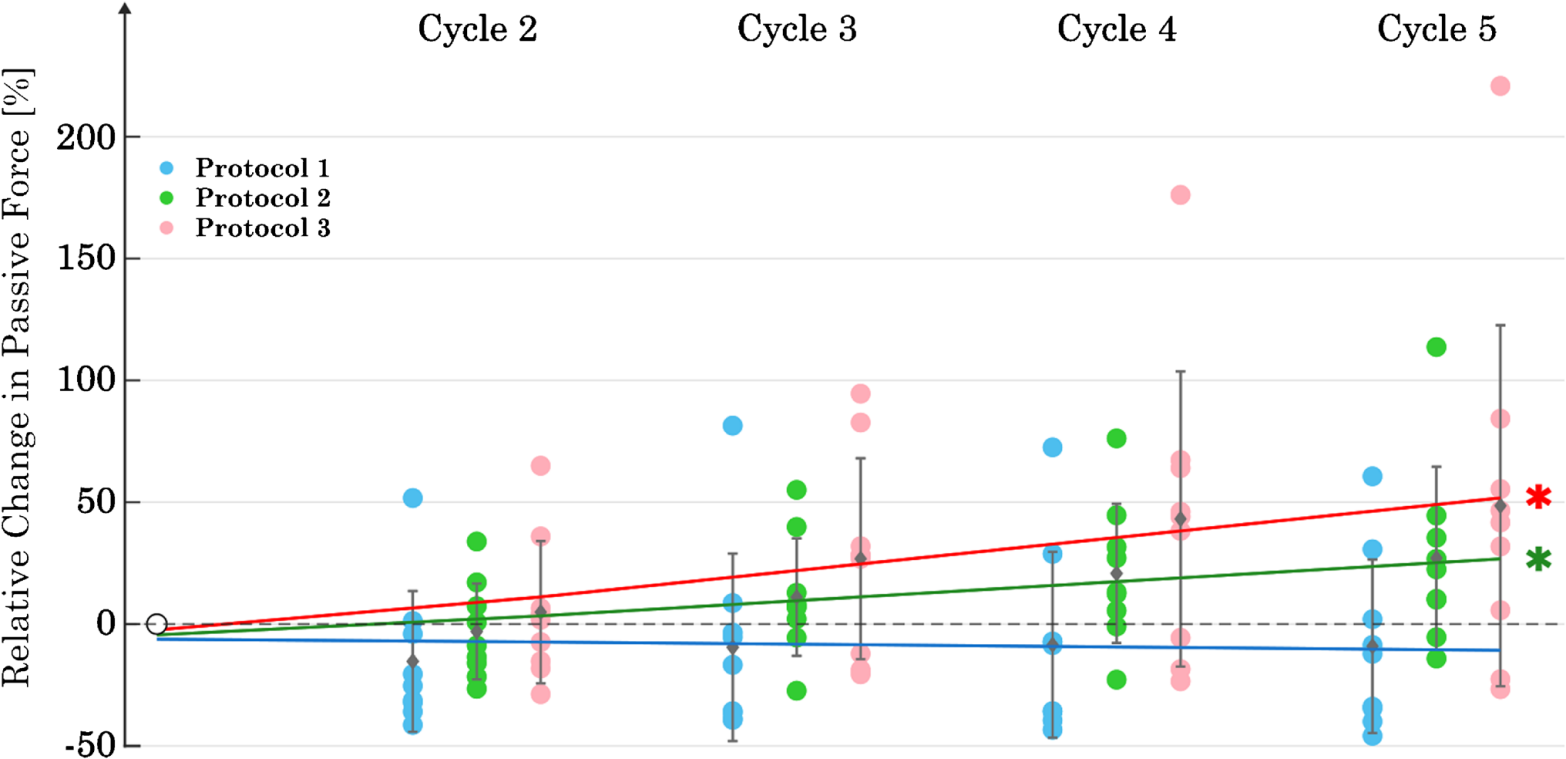
Changes in passive forces at λ = 2 over 5 cycles for each of the three protocols (*n* = 9). The data have been normalized to the value of the first cycle. The asterisk symbol (*) indicates a statistically significant difference (*p* < 0.05) in the regression analysis within each protocol for the slope from C_1_ to C_5_

For P2 and P3, the passive forces increased with each cycle (Fig. 5). The mean nominal passive stress during C_1_ was 5.6 ± 3.1 kPa for P1, 4.2 ± 1.6 kPa for P2, and 4.6 ± 2.1 kPa for P3. Regression analysis showed a significant increase in slope for both P2 (*p* = 0.002) and P3 (*p* = 0.001). However, no significant trend in slope was observed for P1 (*p* > 0.05). The ANOVA showed no significant difference between the protocols at C_5_.

**Fig. 6:**
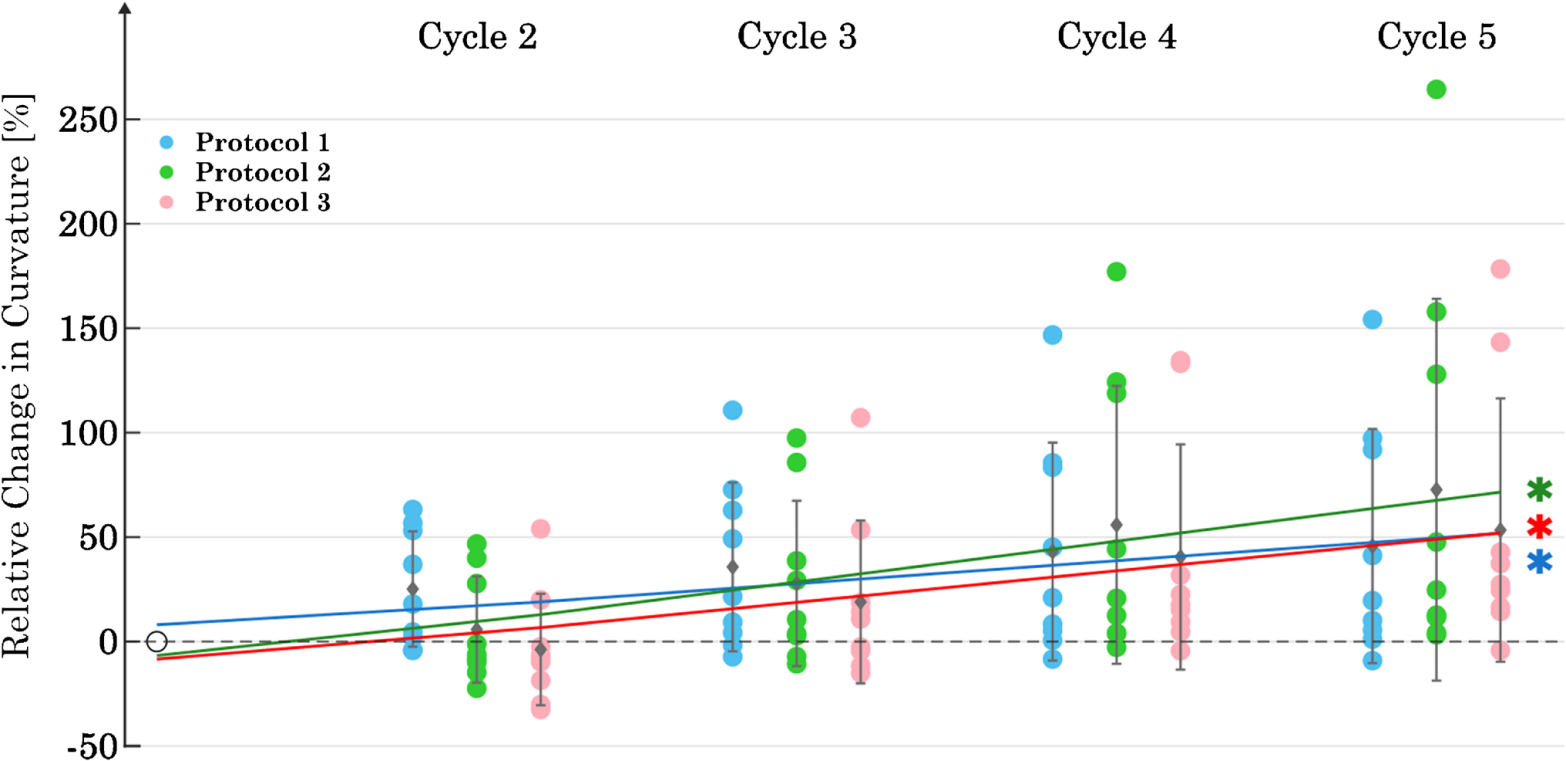
Change in curvature of the stress–strain curves over 5 cycles for each of the three protocols (*n* = 9). The data were normalized to the value of the first cycle. The asterisk symbol (*) indicates a statistically significant difference (*p* < 0.05) in the regression analysis within each protocol for the slope of C_1_ to C_5_

For all three protocols, there is an increase in curvature from C_1_ to C_5_ (Fig. 6). An increase in curvature in this case is equivalent to an increase in stiffness at the end of the lengthening ramp. The mean absolute value at C_1_ was 0.5 ± 0.3 [ −] for P1, 0.4 ± 0.2 [ −] for P2, and 0.4 ± 0.2 [ −] for P3. Regression analysis showed a significant increase in slope for all three protocols (P1: *p* = 0.01; P2: *p* = 0.001; P3: *p* = 0.002). The ANOVA showed no significant differences between the protocols at C_5_.

## Discussion

The aim of this study was to find a method to reproduce the active and passive force response in SM tissue constantly over five repeated cycles of muscle stimulation at long lengths, thus reversing the induced SM adaptation. Three protocols were developed for this purpose, differing in the number of additional stimulations at a short muscle length (P1: 0, P2: 1, P3: 2 stimulations).

The results show that the lengthening ramp and the subsequent isometric contraction induced an adaptation in *F*_act_. This is evidenced by the significant increase (68%) in *F*_act_ (*p* < 0.05) in P1 over the course of the five cycles. The reproduction of *F*_act_ was achieved in both P2 and P3, with the regression lines showing no significant change in *F*_act_ over five cycles (*p* > 0.05 in both cases). Although the passive forces in the final cycle did not differ between the three protocols, only P1 showed no significant change in *F*_pass_ over the five cycles. Consequently, a change in the passive forces was induced by P2 and P3, and the design of P1 appears to be the most suitable for keeping *F*_pass_ constant over five cycles. None of the protocols were able to reproduce the curvature of the stress–strain response over five cycles.

### Comparison with literature

A comparison with the existing literature is difficult due to the lack of comparable protocols in previous studies. Nevertheless, a qualitative comparison with selected studies on muscle adaptivity is presented here.

Wang et al. [48] showed an increase in *F*_act_ with multiple contractions of the same length. This is consistent with the results obtained for protocol 1 (Fig. 4). Speich et al. [40] reported an increase in *F*_pass_ after inducing contractions at short lengths on previously stretched tissue. This observation is consistent with our results for protocols P2 and P3 (Fig. 4), where stimulations were also performed at short muscle lengths. Furthermore, our results for increasing the curvature of stress–strain curves with increasing number of passive stretches (Fig. 6) align with the results of previous studies on rabbit detrusor muscle [39] and porcine bladder [27]. Both studies demonstrate a significant increase in curvature from the first to the second cycle, which persists with each additional cycle, although to a lesser extent. The change in curvature appears to depend mainly on passive lengthening, as there was no discernible effect of the number of activations (P1, P2, P3) on the slope of the regression line (Fig. 6). An increase in curvature is associated with a decrease in the area under the stress–strain curve and thus a loss of stored energy in the passive elements [39]. According to Speich et al. [39], the tissue reaches a viscoelastic steady state after approximately seven to eight repeated stretch ramps. This explains the continuous increase in curvature observed during the five ramps in our data.

Both Gazzola et al. [12] and Lee-Gosselin et al. [23] reported a clear relationship between SM length and force development. A 30% increase in airway SM length resulted in an increase in total force (*F*_tot_) of up to 117.8% [23]. In our results, *F*_act_ for P1 increased by an average of ~ 60% after 5 cycles at the same strip length (λ = 2), while *F*_pass_ remained unchanged. The influence of different strip lengths on the relative changes in muscle force during repeated activations at short lengths remains to be investigated.

### Adaptivity mechanisms

**Fig. 7:**
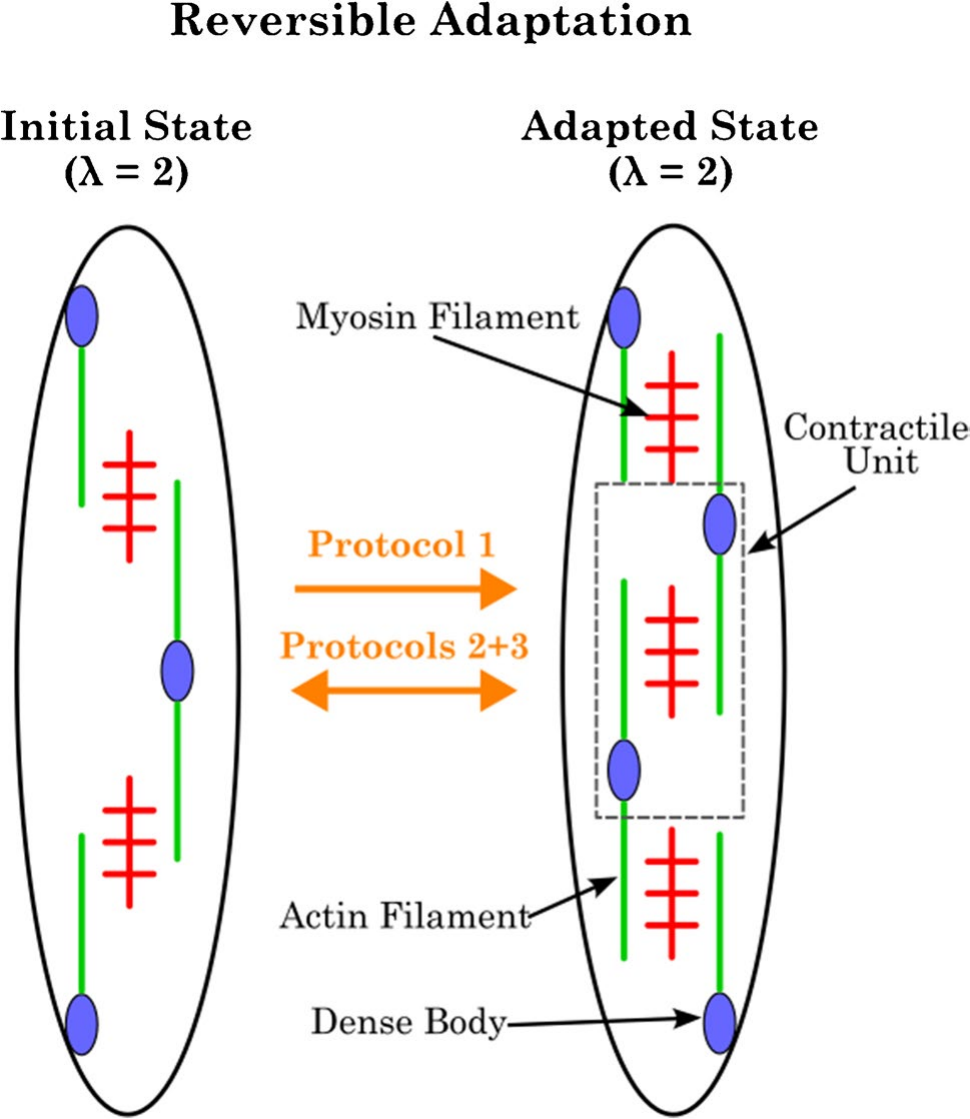
Schematic representation of the model proposed by Wang et al. [46] for the reversible adaptation processes within the contractile apparatus of SMCs. The gray square represents a contractile unit. When a SM is first stretched (initial state) to λ = 2, the myosin and actin filaments do not have an optimal overlap. By inducing contractions to the muscle tissue at the longer length, additional contractile units are added to the contractile apparatus to restore the optimal overlap of the myosin and actin filaments. With the protocols P2 and P3 (Fig. 2), this process is reversible, with contractile units being built out. Adapted from Kuo et al. [22] and Wang et al. [46]

Kuo et al. [22] and Bossé et al. [4] propose that changes in SM length lead to alterations in the contractile apparatus, which in turn affect the active and passive forces of the tissue. According to this hypothesis, in order to adapt to longer lengths, contractile units (sarcomeres) are added to the contractile apparatus. Conversely, to adapt to shorter lengths, contractile units are removed from the array (Fig. 7). This enables the SM tissue to maintain an optimal overlap of actin and myosin filaments for cross-bridge cycling at different lengths, which explains the shift in the force–length relationship after length adaptation [19, 48]. The underlying mechanism for these structural changes remains largely unknown. However, Chitano et al. [8] found evidence that myosin monomers are recruited into the filament lattice during length adaptation. The authors describe two myosin pools within the SMC: the monomeric myosin (non-muscle myosin) and the filamentous myosin (SM myosin). The process of length adaptation now alters the equilibrium between the two pools.

This model provides an explanation for the observed changes in *F*_act_ for the three different protocols. For P1, each ramp, in combination with the isometric contraction at λ = 2, induces the installation of additional myosin filaments in series and in parallel [19, 22]. *F*_act_ could be increased by a better overlap of myofilaments, longer myosin filaments, or more contractile units in parallel [19, 41]. Shortening the tissue to λ = 1, followed by an isometric contraction, results in the removal of thick filaments (or impedes the previous induced installation of more myosin). This does not result in an increase in *F*_act_ with each cycle; rather, it maintains the same level (see P2). The results of this study (Fig. 3) indicate that increasing the number of contractions at the shortened length from one (at P2) to two (at P3) does not result in any additional effect on the removal of thick filaments.

**Fig. 8:**
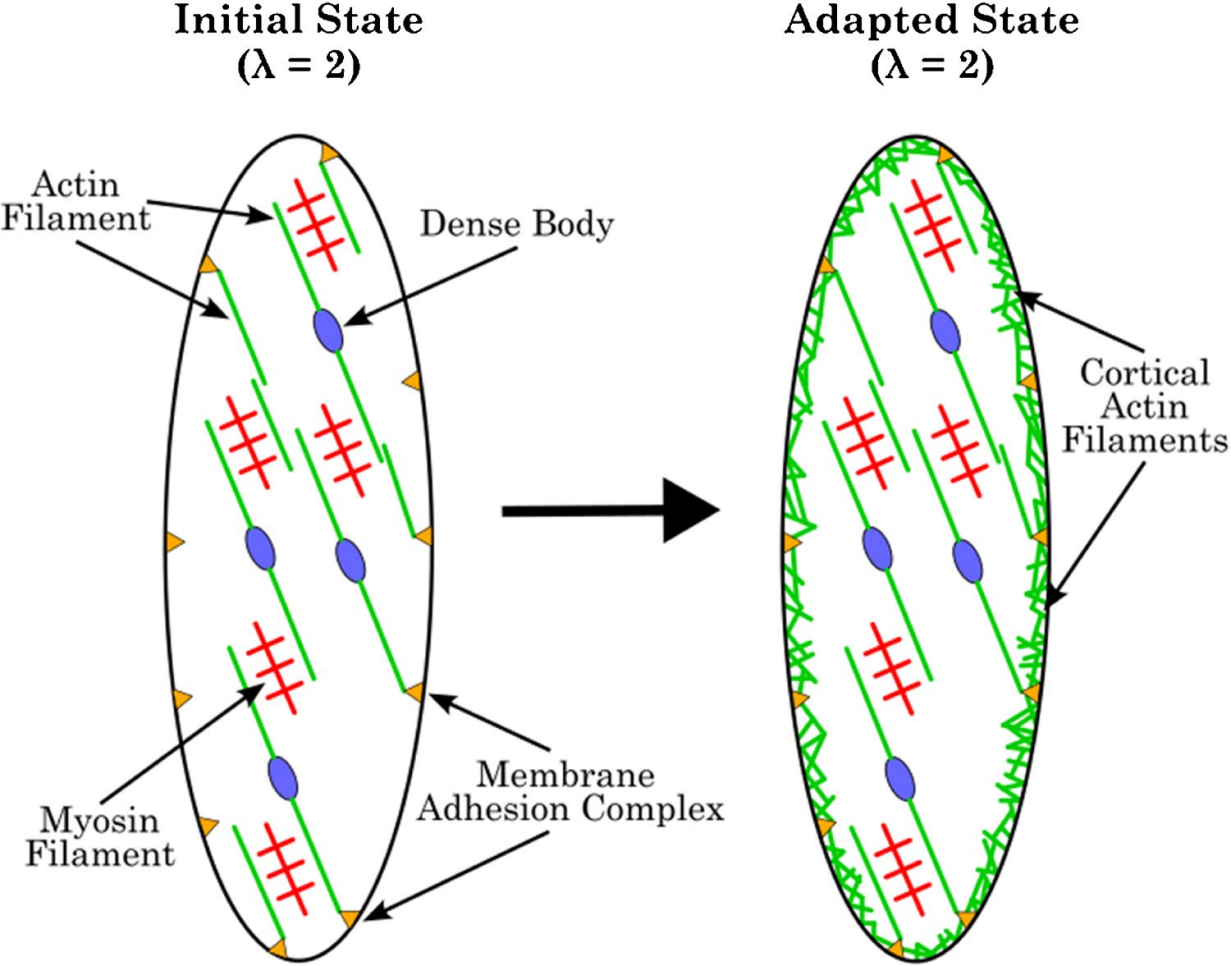
Schematic illustration of actin-specific adaptation processes in SMCs in response to contractile and mechanical stimuli. Both conditions initiate a molecular cascade via the membrane adhesion complexes, which ultimately lead to actin polymerization at the cortical region of the cell. Subsequently, the polymerized actin is utilized for cytoskeletal rearrangements, which ultimately result in cell stiffening. Adapted from Gunst and Zhang [17]

While the malleability of the actomyosin complex appears to influence the development of active forces, there is evidence in the literature that the cytoskeleton, with its integrated actin filaments, also plays an important role in the development of mechanical tension in the SMC. As with myosin, there are pools of free globular actin as well as filamentous actin [13, 52, 53]. Gazzola et al. [13] showed that SM activation alters the ratio of these two actin pools, as is the case with myosin. Other studies have provided evidence that not only activation [25] but also passive stretch [10] induces processes that alter the cytoskeleton of SMCs, including the actin filament lattice. Both stimuli activate a cascade of molecular processes via the so-called adhesion complexes, resulting in the polymerization of actin filaments and corresponding remodeling of the cytoskeleton in the peripheral region (e.g., cortical actin filaments) of the cell (Fig. 8) [16, 17, 36, 53]. The formation of this cortical actin-cytoskeleton structure in response to stretch or activation increases the stiffness of the cell. This remodeling is beneficial for the transmission of cross-bridge forces to the outside of the cell and ultimately results in increased stiffness and passive forces throughout the SM tissue [17].

The onset of cytoskeletal remodeling processes towards increased stiffness seems to be independent of the direction of mechanical stretch and the length of the contractions. In the case of protocols P2 and P3, it can be reasonably concluded that with each additional contraction, the cortical polymerization of actin filaments was induced, thereby increasing the stiffness of the cell or muscle tissue. This is reflected in the increasing *F*_pass_ for P2 and P3 (Fig. 5). Since P3 contains one additional contraction compared to P2, it is evident that P3 has the highest *F*_pass_ after five cycles (Fig. 5). As proposed by Yamin and Morgan [50], depolymerization is likely to occur during relaxation to reverse the cytoskeletal stiffening that has occurred during the contraction phase. However, this effect has been little studied.

### Limitations

The protocols presented in this study do not resemble the physiological behavior of bladder tissue. Physiologically, SMCs are relaxed during the filling phase and contractions occur only at long lengths, when micturition is induced in a full bladder [1]. The stimulation duration of 20 s is within the physiological micturition duration, which is 21 ± 13 s for all mammals over 3 kg body weight [51]. The protocols presented here are able to control muscle adaptation by introducing rest periods and short-length activation. In future studies, these steps can be incorporated into experimental protocols on SM tissue where adaptation processes would otherwise alter mechanical properties.

## Conclusion

The aim of this study was to develop experimental protocols to reproduce the initial mechanical properties of SM tissue by inducing and reversing adaptation processes through the application of stretches and contractions of different lengths. The protocols developed allowed the reproduction of an initial active force at long lengths by adding contractions at short lengths (P2 and P3). However, the same protocols failed to reproduce the initial passive forces. Conversely, the passive forces increased with each cycle. Furthermore, the increase in curvature over five cycles indicates the loss of stored energy in the passive cell elements. The observed behavior of both active and passive forces can be explained by existing theories in the literature for the adaptation of SM tissues. Active force is primarily influenced by the addition or removal of contractile units to and from the contractile apparatus, whereas changes in passive force are mainly due to the contraction-induced actin polymerization and consequent cytoskeletal stiffening. The question of actin depolymerization and subsequent recovery of the initial passive forces remains open for further investigation.

## Data Availability

The datasets generated during and/or analysed during the current study are available from the corresponding author on reasonable request.
